# Description of a new species of *Gaeolaelaps* (Acari: Laelapidae) from Iran

**DOI:** 10.3897/zookeys.612.9678

**Published:** 2016-08-23

**Authors:** Zarir Saeidi, Alireza Nemati, Arsalan Khalili-Moghadam

**Affiliations:** 1 Department of Plant Protection, Agricultural and Natural Resources Research and Education Center, Chaharmahal va Bakhtiari; 2 Plant Protection Department, Agricultural College, Shahrekord University, Shahrekord, Chaharmahal va Bakhtiari, Iran

**Keywords:** Chaetotaxy, Mesostigmata, mite, soil, taxonomy

## Abstract

A new species of *Gaeolaelaps* (Acari, Mesostigmata, Laelapidae), *Gaeolaelaps izajiensis* **sp. n.** is described based on the morphological characters of adult females which were collected from soil sample in the Izeh and Ghaletol regions of the Khuzestan province, Iran. It can be distinguished from the other members of the genus by some morphological characteristics of dorsal shield, form and reticulation of epigynal shield, the exopodal plates, and the peritremes.

## Introduction

Mites of the family Laelapidae are ecologically divers and comprise parasites and predators which found in various habitats (Strong and Halliday 1994, Beaulieu 2009, Lindquist et al. 2009, Nemati and Mohseni 2013). They are good candidates for biological control of the pests which spend time in the soil or other plant growing media (Beaulieu 2009). The family increased in the size with around 90 known genera and more than 1300 species (Beaulieu et al. 2011). *Gaeolaelaps* is a large cosmopolitan genus of the Laelapidae family which consists of more than 100 described species (Nemati and Mohseni 2013, Kazemi et al. 2014). Different types of the habitats were reported for the *Gaeolaelaps* mites including: soil, litter, nests and bodies of vertebrates and invertebrates (Bregetova 1977, Beaulieu 2009, Lindquist et al. 2009, Trach 2012). *Gaeolaelaps* species are typically known as small invertebrate predators, and collected from the bodies and nests of many arthropods including cockroaches, termites, mole crickets, beetles, ants, millipedes and mygalomorph spiders (Bregetova 1977, Rosario 1981, Tenorio 1982, Strong and Halliday 1994, Fain et al. 1995, Strong 1995, Beaulieu 2009, Faraji and Halliday 2009).

Twenty species of *Gaeolaelaps* have been reported from Iran of which nine were described as new for science (Nemati and Kavianpour 2013, Nemati and Mohseni 2013, Kavianpour et al. 2013, Kazemi et al. 2014, Kavianpour and Nemati 2014, Vatankhah et al. 2016). It is noticeable that the majority of these species (seven species) have been collected from soil (Nemati and Kavianpour 2013, Nemati and Mohseni 2013, Kavianpour et al. 2013, Kazemi et al. 2014, Kavianpour and Nemati 2014), one species (Vatankhah et al. 2016) has been described from the nest of *Formica* sp. (Hymenoptera: Formicidae) and one species from the body of a carabid beetle, *Acinopus* sp. (Coleoptera: Carabidae) which the later had been previously excluded from *Gaeolaelaps* by Kazemi et al. (2014). Four of these species including *Gaeolaelaps farajii* Nemati & Mohseni, 2013; *Gaeolaelaps jondishapouri* Nemati & Kavianpour, 2013; *Gaeolaelaps khajooii* Kazemi, Rajaei & Beaulieu, 2014 and *Gaeolaelaps orbiculatus* Nemati & Mohseni, 2013 were reported from the south of Iran (Khuzestan and Kerman provinces) while one species (*Gaeolaelaps ahangarani* Kazemi & Beaulieu, 2014) has been described from the north (Mazandaran province) and three species (*Gaeolaelaps iranicus* Kavianpour & Nemati, 2013, *Gaeolaelaps mossadeghi* Kavianpour & Nemati, 2013 and *Gaeolaelaps lenis* Vatankhah & Nemati, 2016) from the central part of Iran (Esfahan province). Here a further new species is described, which will be the tenth species from Iran; it was collected from the soils of Izeh and Ghaletol, Khuzestan province, Iran.

## Materials and methods

*Gaeolaelaps* specimens were extracted from soil samples using Berlese funnels, placed in lactic acid at 55 °C for clearing and then mounted in Hoyer’s medium as permanent microslides for microscopic examination. Taxonomically relevant structures of this species were illustrated with the use of a drawing tube and figures were performed with Corel X-draw software, based on the scanned line drawings. Measurements of structures are expressed as minimum-maximum ranges in micrometers. The dorsal setae notation, leg and palp chaetotaxy follows that of Lindquist and Evans (1965), Evans (1963a, b) and Evans and Till 1965 respectively. Terminology for idiosomal glands and lyrifissures follows Kazemi et al. (2014). Legs were measured dorso-medially excluding the stalk and pretarsus.

## Reults

### Genus Gaeolaelaps

Taxon classificationAnimaliaMesostigmataLaelapidae

Evans & Till, 1966

Hypoaspis (Gaeolaelaps) Evans & Till, 1966: 160; Evans and Till 1979: 202.
Hypoaspis (Geolaelaps) : Bregetova 1977: 499; Karg 1979: 79; Karg 1982: 237; Karg 1993: 136.
Gaeolaelaps : Casanueva 1993: 40; Beaulieu 2009: 35; Kazemi et al. 2014: 504.
Geolaelaps : Rosario 1981: 46; Walter and Oliver 1989: 295.

#### Type species.

*Laelaps aculeifer* Canestrini (1884), by original designation (Evans and Till 1966).

The genus definition of Kazemi et al. 2014 was followed (see notes in discussion).

### Gaeolaelaps izajiensis sp. n.

Taxon classificationAnimaliaMesostigmataLaelapidae

http://zoobank.org/F8E6016A-33DA-4B04-97FB-67092192A3A9

Figures 1–5 , 6–9 , 10–13

#### Specimens examined and type deposition.

Holotype female, Izeh, Khuzestan province, soil, coll. A. Nemati, 2013. Paratypes: two females, Ghaletol, Khuzestan province, soil, coll. Z. Saeidi, 2014. The holotype and two female paratypes are deposited in the Acarological Laboratory, Department of Plant Protection, Agricultural College, Shahrekord University, Shahrekord, Iran (APAS).

#### Diagnosis (adult female).

Dorsal shield with constriction at lateral margins near setae s6 and distinct reticulation posterior to *j6* along with line reticulation in lateral margins of podonotal part, possesses 39 pairs of simple thin acicular setae; sternal shield with reticulation in lateral regions, epigynal shield with elongate and nearly quadrangle cells and abutting anal shield, exopodal plates fragmented between coxae II and IV; peritremes relatively long and extending to the posterior margin of coxae I.

#### Description of adult female.

Three specimens measured, range is provided in µm.

**Figures 1–5.**
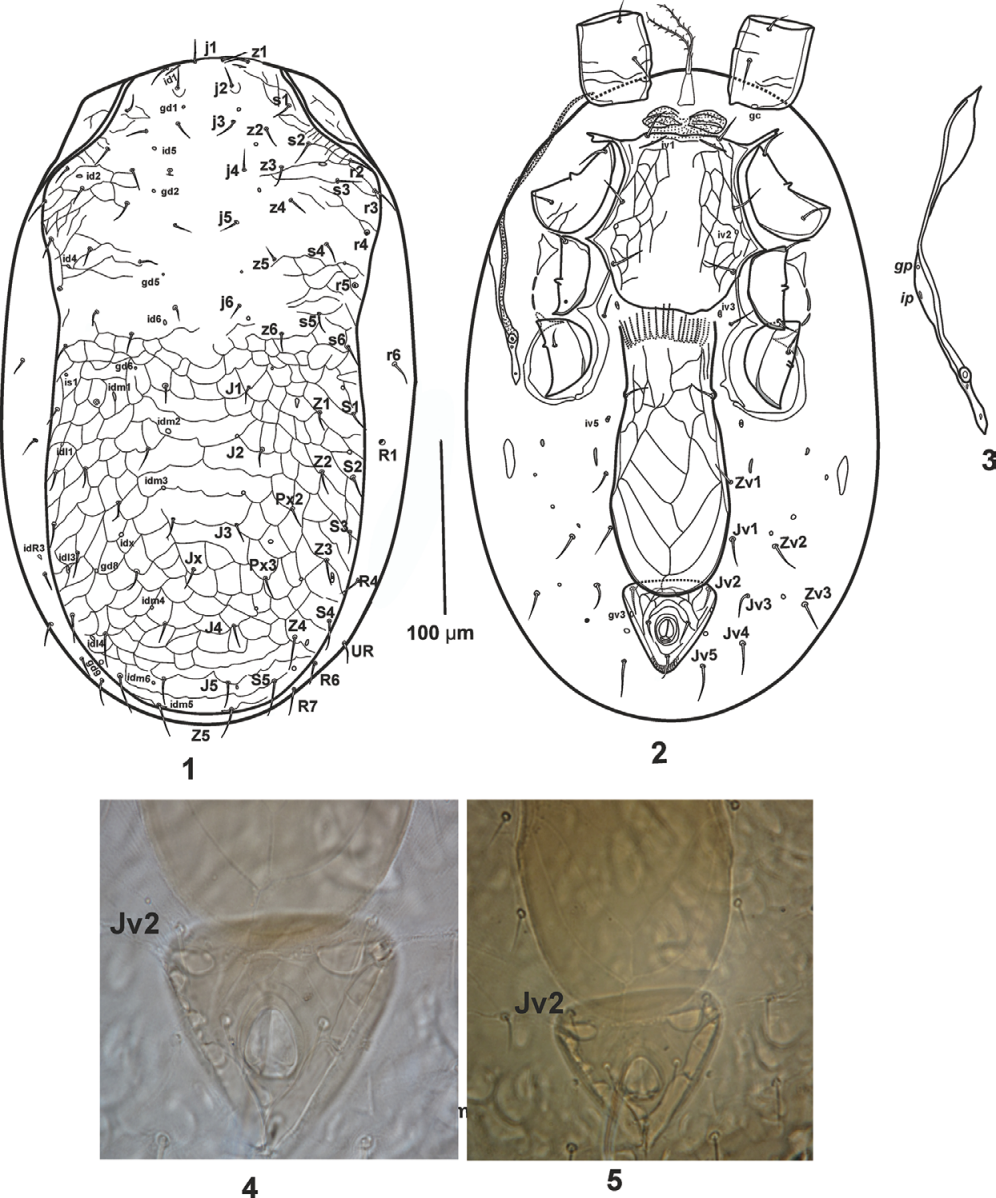
*Gaeolaelaps izajiensis* sp. n. Female: **1** dorsal idiosoma **2** ventral idiosoma **3** Peritreme and sub-triangular accessory shield **4–5** the position of *Jv2*.

***Dorsal idiosoma*.** Idiosoma oval-shaped 431–442 long, 266–273 wide (at level of setae *r3*), dorsal shield with constriction at lateral margins near *s6* setae and distinct reticulation posterior to *j6* along with line reticulation in lateral margins of podonotal part, shield not covering whole dorsum (Fig. 1), 400–419 long from its anteromedian edge anterior to bases of setae *j1* to its posteromedian edge posterior to bases of setae *Z5*, 240–247 wide at level of setae *r2-r3* (widest part), shield with 39 pairs of thin, small, simple acicular setae, 22 pairs on podonotal region (*j1–6*; *z1–6*; *s1–6*; *r2–5*) and 17 pairs on opisthonotal part (*J1–5*; *Z1–5*; *S1–5*) including *PX2–3* between *J* and *Z* series. Unsclerotised cuticle lateral of podonotal region including a smooth sub-triangular accessory shield (Fig. 1). Dorsal setae short (22–30), not reaching to following seta base in series. Setae *J3* located nearly far from *J4*, the distance of *J3-J4* is approximately five times *J3* length. Unsclerotised cuticle lateral of podonotal part with *r6* (between *s6* and *S1*) and lateral of opisthonotal with *R1*, *R4*, *R6* and *R7*. *UR* seta located between *R4* and *R6*. Dorsal shield with 22 pairs of pores and pore-like structures, including *gd2* (posterolaterad of setae *j4*) and *gd6* (posterior of *z6*) and one pair of poroids (*idR3*) on soft lateral cuticle near *R4* seta as shown in Figure 1.

***Ventral idiosoma*** (Fig. 2). Base of tritosternum 25–30 long, 7–10 wide (at basal level), pilose laciniae free for 59–73 and fused basally for 7–9. Pre-endopodal area granulated, with a pair of slightly sclerotised pre-sternal plates. Sternal shield 120–127 long (along midline from anterior edge to its posterior margin), 98–110 wide (at level of projection between coxae II-III) and 81–88 at level of *st2*, smooth in median region and posterior part and reticulated in lateral margins, with distinct anterior and posterior margins, posterior margin irregular. Sternal setae smooth, *st1- st3* (20–23), *iv1* slit-like, located slightly behind *st1*, *iv2* pore-like, between *st2*-*st3*. Setae *st4* (16–19) and pore-like *iv3* located on integument behind posterior margin of sternal shield. Reticulate tongue-shaped epigynal shield with elongate and nearly quadrangle cells, 171–180 long at midline from anterior margin to posterior level and abutting anal shield, 73–83 wide at epigynal setae, ratio of length to width (L/W) 2.14–2.4, with one pair of simple acicular setae (*st5* = 18–20). Paragenital pores (*iv5*) on soft integument posterior to epigynal setae, between epigynal margin and coxa IV. Anal shield subtriangular, reticulated, 54–60 long (at midline from the anterior margin to the posterior edge of the cribrum), 54–56 wide (at widest point), post anal seta (14–15) nearly equal to para-anal setae (15–17). Cribrum thin and extending posterolaterally to the level of post-anal seta insertion. Opisthogastric surface with: one pair of narrow and slightly elongate paragenital platelets; one pair of suboval metapodal plates (24–26×10–12); one pair of minute platelets between paragenital and metapodal plates; eight pairs of smooth acicular setae: *Zv1*–3 and *Jv1–5*; and five pairs of pore-like structures, plus para-anal gland pores *gv3* on lateral margins of anal shield. *Jv2* located on soft opisthogastric cuticle at postero-lateral part of epigynal shield (Fig. 4). In holotype this part of soft cuticle bent down and *Jv2* appeared on anterior margin of anal shield as shown in Figures 2 and 5. Stigma located at anterior level of coxa IV. Peritremes narrow and long, extending anteriorly to posterior margin of coxae I with fusing at posterior margin of sub-triangular accessory shield, peritrematal plate wider in middle part, and with one glandular poroid *gp* and one lyrifissure *ip* (Fig. 3), separated from exopodal shield. Poststigmatal plate narrow and with two pore-like structures. Exopodal II-III small and subtriangular, along with two fragmented platelets at posterior part of coxa III. Exopodal III-IV narrow, angular and reached to the tip of endopodal III-IV at posterior level of coxa IV. Endopodal plates II/III incorporated to lateral margins of sternal shield, III-IV strip like extending to the posterior margin of coxa IV.

**Figures 6–9.**
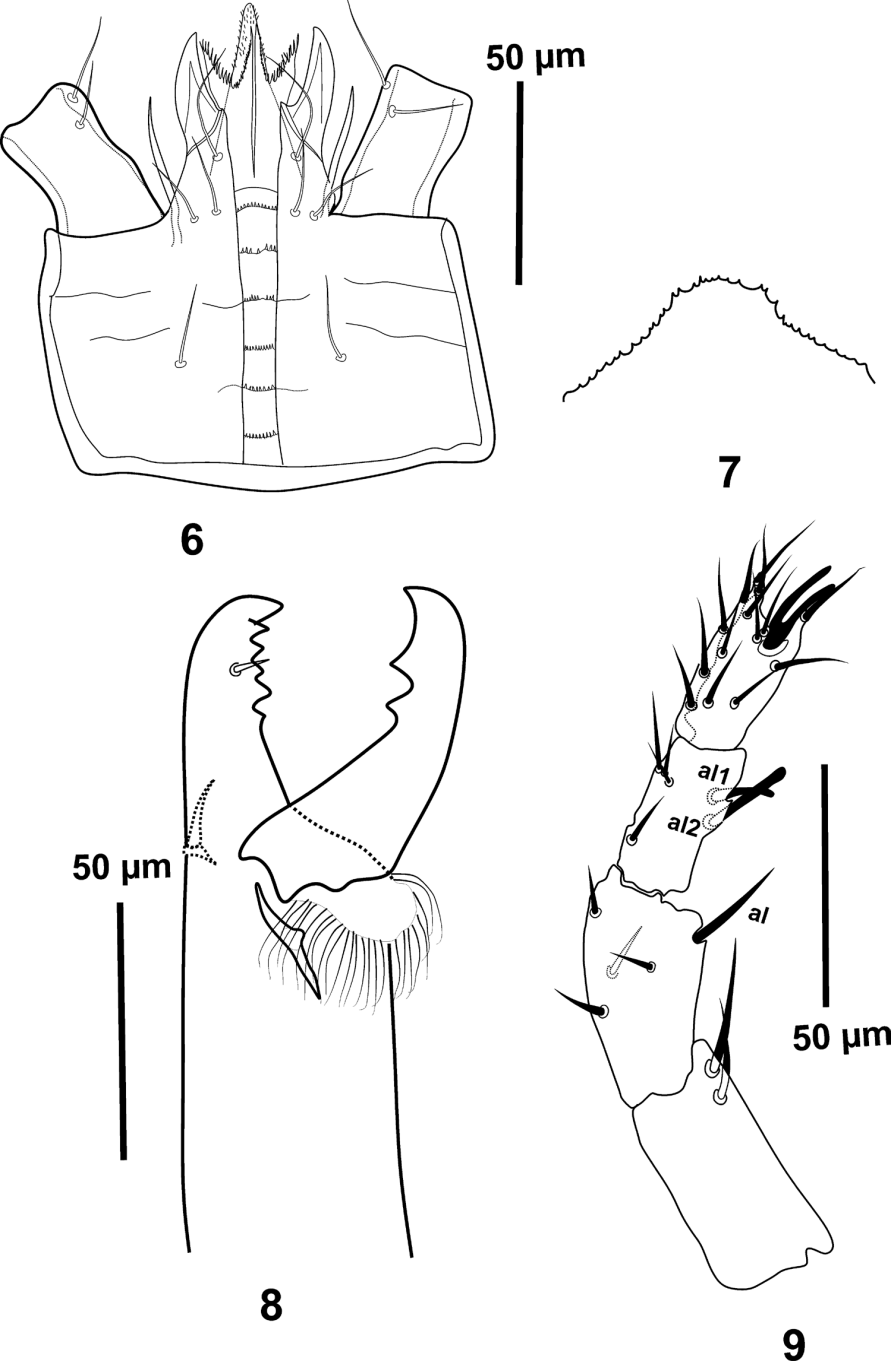
*Gaeolaelaps izajiensis* sp. n. Female: **6** hypostome **7** epistome **8** chelicera **9** palp (trochanter to tibia).

***Gnathosoma*.** Hypostome (Fig. 6) with three pairs of smooth simple acicular setae; *h1* (28–32), *h2* (15–17) and *h3* (17–19). Palpcoxal setae 17–19 long. Deutosternal groove with six rows of denticles (7–12). Corniculi horn-like, internal malae with median barbed extensions longer than fringed lateral lobes, labrum short and slightly pubescent. Epistome denticulate (Fig. 7). Chelicera (Fig. 8) with dorsal seta, small and setaceous pilus dentilis, lateral lyrifissure and arthrodial crownet-shaped, moveable digit (46–54) with two teeth; middle article from the basal level to the base of dorsal seta 78–85 ending in fixed digit (49–51) with five teeth in addition to terminal tooth. Palp chaetotaxy normal for Laelapidae mites (sensu Evans & Till, 1965), with simple setae except *al1* and *al2* of genu slightly thickened with blunt tip, palp apotele two-tined (Fig. 9).

***Legs*.** Tarsi I-IV with claws and ambulacra. **leg I** 427–432, coxa 61–63, trochanter 39–44, basi-femur 20–24, telo-femur 61–68, genu 66–73, tibia 78, tarsus 90–95; **leg II** 317–329, coxa 37–39, trochanter 41–44, basi-femur 17–22, telo-femur 49–56, genu 46–54, tibia 46–49, tarsus: 73–78; **leg III** 249–259, coxa 22, trochanter 37–41, basi-femur 15–20, telo-femur 39, genu 24–32, tibia 37–41, tarsus 63–73; **leg IV** 383–417, coxa 34–37, trochanter 68–73, basi-femur 20–26, telo-femur 54–61, genu 49–56, tibia 54–61, tarsus 98–103. Legs I and IV longer than legs II and III. Chaetotaxy of all leg segments normal for *Gaeolaelaps* (sensu Faraji & Halliday, 2009). All leg setae smooth and pointed.

**Figures 10–13.**
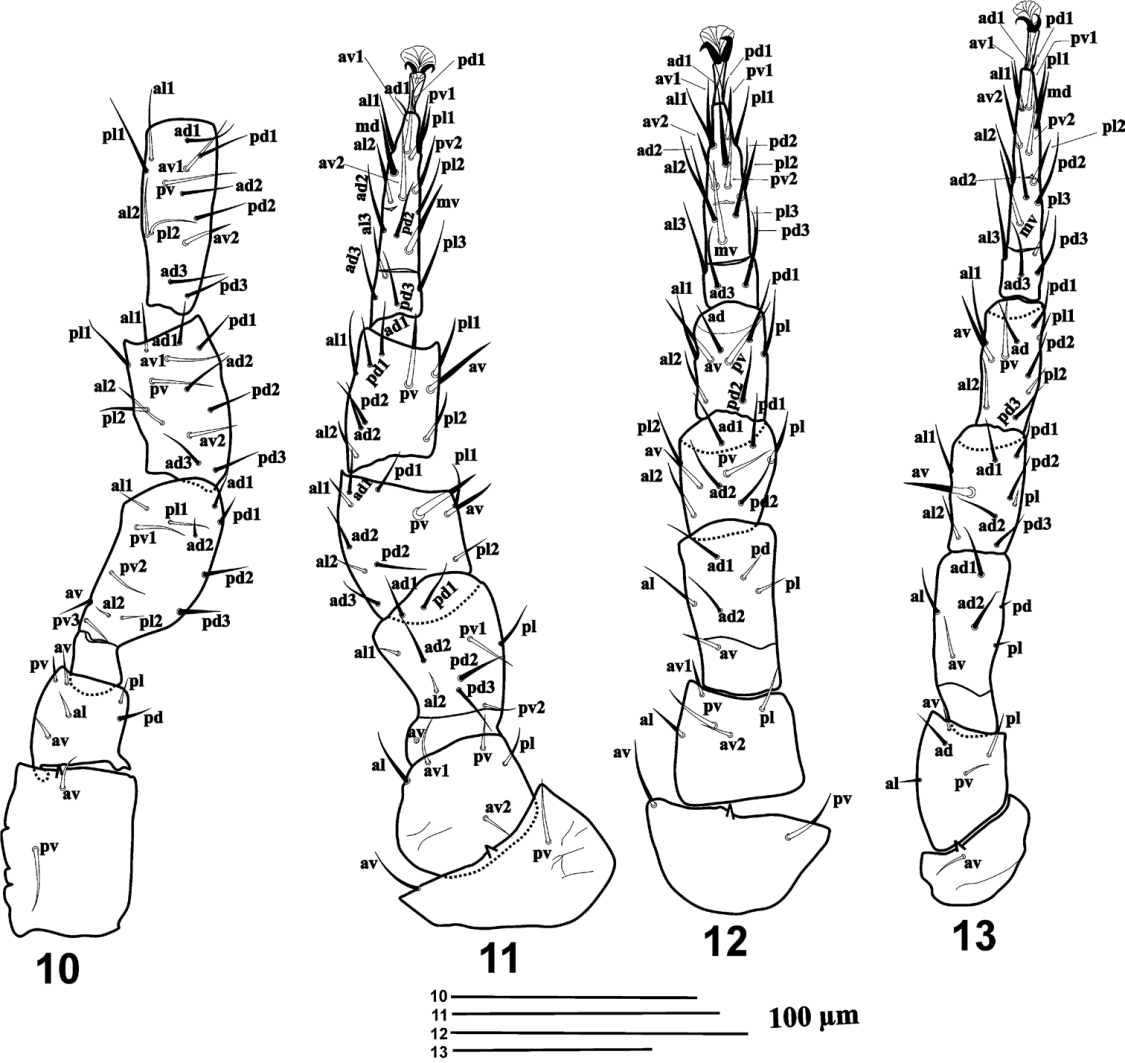
*Gaeolaelaps izajiensis* sp. n. Female: **10** Leg I **11** Leg II **12** Leg III **13** Leg IV.

***Legs Chaetotaxy*** (Figs 10–13): **Leg I** (Fig. 10): coxa 0 0/1 0/1 0; trochanter 1 0/2 1/1 1; femur 2 2/1 3/3 2; genu 2 3/2 3/1 2; tibia 2 3/2 3/1 2. **Leg II** (Fig. 11): coxa 0 0/1 0/1 0; trochanter 1 0/2 0/1 1; femur 2 3/1 2/2 1; genu 2 3/1 2/1 2(*pv* slightly thicker than other setae on the segment); tibia 2 2/1 2/1 2 (*av* and *pv* slightly thicker than other setae on the segment); tarsus 3 3/2 3/2 3 + *mv*, *md* (*pl1*, *al1*, *pv1*﻿﻿﻿–*2*, *av1–2*, *md* and *mv* slightly thicker than other setae on the segment). **Leg III** (Fig. 12): coxa 0 0/1 0/1 0; trochanter 1 0/2 0/1 1; femur 1 2/1 1/0 1; genu 2 2/1 2/1 1; tibia 2 1/1 2/1 1; tarsus 3 3/2 3/2 3 + *mv*, *md* (the thickness of setae similar to those on tarsus II). **Leg IV** (Fig. 13): coxa 0 0/1 0/0 0; trochanter 1 0/2 0/1 1; femur 1 2/1 1/0 1; genu 2 2/1 3/0 1 (*av* thicker than other setae on segment); tibia 2 1/1 3/1 2 (*av* and *pv* slightly thicker than other setae on the segment); tarsus 3 3/2 3/2 3 + *mv*, *md* (*av1–2*, *pv1–2*, *pl2*, *mv* and *md* slightly thicker than other setae on the segment. All setae fine and needle-like unless otherwise noted.

***Insemination structures*.** Not seen.

#### Male.

Unknown.

#### Etymology.

The name of the new species refers to Izaj, the ancient name of Izeh (a town in Khuzestan province, southwest Iran) where the holotype was collected.

#### Remarks.

*Gaeolaelaps izajiensis* sp. n. is differentiated from all other members of the genus by the following combination of characters: dorsal shield with constriction at lateral margins near setae s6, with 39 pairs of simple thin acicular setae; reticulated epigynal shield with elongate and nearly quadrangle cells and abutting anal shield, exopodal plates fragmented between coxae III and IV; peritremes long and extending to the posterior margin of coxae I. Some species of *Gaeolaelaps* genus have long epigynal shield like: *Gaeolaelaps loksai* (Karg, 2000), *Gaeolaelaps pinnae* (Karg, 1987) and *Gaeolaelaps macra* (Karg, 1978) in which the epigynal shield extending near anal shield with only one pair of opisthogastric setae between epigynal and anal shields. *Gaeolaelaps macra* and *Gaeolaelaps loksai* have short peritremes which extended to near anterior and middle level of coxa II, respectively. *Gaeolaelaps pinnae* has long peritreme extending to anterior part of coxa I but has long dorsal setae which exceed the base of successive setae in series, seta *J1* and some other opisthonotal setae barbed distally and *iv2* slit-like (Karg 1978, 1987, 2000).

## Discussion

*Gaeolaelaps* has been defined in details by Beaulieu (2009) and Kazemi et al. (2014). The new species described in this paper is well accordance with definition of Kazemi et al. (2014) except for the characteristic no. 10 (p. 504). They stated that epigynal shield tongue or flask-shaped, not markedly broadened posteriorly, bearing one pair of simple setae, and not touching anal shield.

In some species of this genus (*Gaeolaelaps loksai* (Karg), *Gaeolaelaps pinnae* (Karg) and *Gaeolaelaps macra* (Karg)) epigynal shield extending near subtriangular anal shield in which there is only one pair of opisthogastric setae between these two shields, but in fact none of *Gaeolaelaps* species has epigynal shield abutting subtriangular anal shield. Our new species has epigynal shield uniquely long and extended to the anal plate with one pair of setae (*Jv2*) located at posterior latero-corners of epigynal shield on unsclerotised cuticle of opisthogasteric area. In this paper, we have followed the definition of Kazemi et al. (2014) to consider our new species as a member of *Gaeolaelaps* with the following modification to the no. 10 characteristic (length of epigynal shield):

*10. Epigynal shield tongue- or flask-shaped, not markedly broadened posteriorly, bearing one pair of simple setae, and not touching anal shield in most of the species except for Gaeolaelaps izajiensis n. sp*.

## Supplementary Material

XML Treatment for Gaeolaelaps

XML Treatment for Gaeolaelaps izajiensis
